# Stereoselective Phosphorylation of d-Ribose as a Driver of Life’s Homochirality

**DOI:** 10.3390/life16050846

**Published:** 2026-05-20

**Authors:** Vladimir M. Subbotin, Gennady Fiksel

**Affiliations:** 1 Arrowhead Pharmaceuticals Inc., Madison, WI 53719, USA; 2 University of Wisconsin, Department of Human Oncology, Madison, WI 53792, USA; 3 University of Wisconsin, Department of Physics, Madison, WI 53706, USA

**Keywords:** liposomes, homochirality, enantioselectivity, permeability, phosphorylation

## Abstract

Life demonstrates remarkable homochirality of its major building blocks: nucleic acids, amino acids, sugars, and phospholipids. Phospholipid bilayer vesicles (liposomes) are formed at the water/air interface from Langmuir layers and contain ribose, a constituent of primordial water. Although the primordial ribose was initially racemic, life, as we know it, is homochiral, with d-ribose and its derivatives as the predominant forms. The phospholipid membrane’s permeability to d-ribose, together with ribose’s interaction with the bilayer’s charged phosphate groups, leads to ribose phosphorylation, yielding d-ribose-5-phosphate. Once inside, the d-ribose-5-phosphate molecules cannot cross the membrane. A similar path also exists for l-ribose, but with a lower rate. Therefore, overall, this process is enantioselective, favoring the buildup of d-ribose over l-ribose. Through liposome fusion, fission, and self-replication, this eventually leads to the Darwinian evolution of these structures and to the conversion of d-ribose-5-phosphate into complex functional molecules, such as ribozymes and RNA, and eventually into DNA, all of which inherit d-ribose’s chirality.

## 1. Introduction

Life demonstrates remarkable homochirality of its major building blocks: nucleic acids, amino acids, sugars, and phospholipids [1,2]. Historically, the issue of life’s homochirality has been explored through various concepts, most notably “The Frozen Accident Theory” by Francis Crick, published in 1968 [3] and later extended by others [4,5,6,7]. Additionally, other concepts were considered, including ab initio homochirality [8] as well as cosmological phenomena of different natures and magnitudes: supernovae explosions [9,10], weak nuclear force [11], asymmetric dark matter [12], circularly polarized light generated in the interstellar medium [13], and the concepts of the “sterile”and the left-handed neutrinos [14]. Despite these efforts, homochirality remains a great mystery, perhaps no less perplexing than the Origin of Life itself [14,15,16,17].

Consider, for example, the observed homochirality of ribose, which appears predominantly in the enantiomeric form of d-ribose, That is to say that modern life consists only of d-ribose and its derivatives, even though the primordial ribose, for example, the kind brought by carbonaceous meteorites, appeared as racemic, containing equal parts of d-ribose and l-ribose [18]. Numerous theoretical and experimental studies [19,20] have shown that, due to the high rate of autocatalytic reactions, even a slight imbalance in the initial concentrations of the two enantiomeric forms of molecules can be amplified over time. But even with a high rate of autocatalytic reactions, the outcome still would strongly depend on the initial concentration disparity—the greater the initial difference, the more likely it is to influence the final result. Clearly, there is a need for a robust, universal mechanism that favors the prevalence of d-ribose.

## 2. Darwinian Evolution of Liposomes

Although it may seem that the evolution of liposomes is unrelated to the homochirality problem, we aim to demonstrate that this is not true—the dynamics of liposome evolution and the factors influencing it are directly connected to the issue of chirality.

Several scenarios of the origin of life, e.g., RNA World and Metabolism First, assume the occurrence of multiple events that must align consecutively in time and space, i.e., appear as a multistage process. However, such staging raises strong skepticism, for example, by Oparin [21] and Orgel [22]. An alternative model, Lipid First [23,24,25], places the origin of life into self-assembling lipid molecules and the formation of liposomes before more complex, information-carrying RNA emerged. This process, through self-replication, fusion, and fission, eventually leads to Darwinian evolution of these simple, spontaneously organized structures.

**Figure 1:**
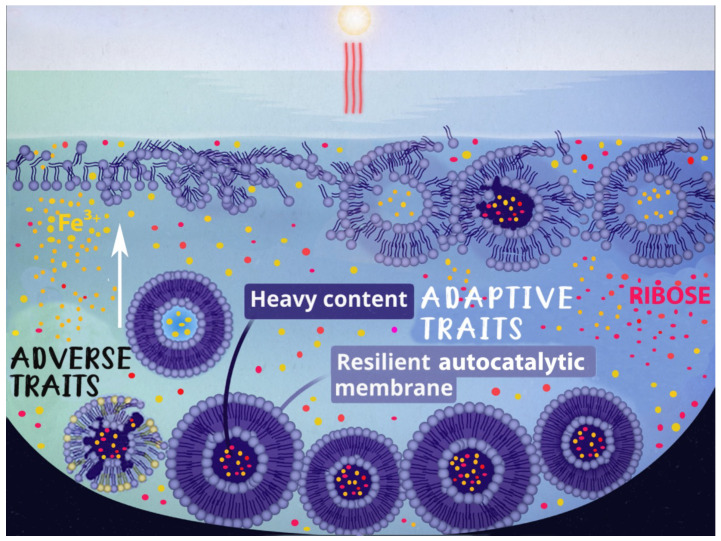
A depiction of liposome formation and evolution. Liposomes formed on the surface of the Archean water pools are destroyed by solar UV unless they acquire two adaptive traits—the heavy content that sinks the liposomes to the bottom of the pool and facilitates protection from UV, and the formation of resilient autocatalytic membrane composition that ensures liposomal survival, fusion, fission, and mutation of the components, thus providing liposomes adaptation and persistence.

We have advanced this concept by proposing a hypothesis of self-sustaining Darwinian evolution of liposomes [26] that relies solely on natural and ubiquitous phenomena: solar UV radiation, the diurnal cycle, and gravity. In our scenario, liposomes formed at the Langmuir layer would be inevitably destroyed by solar UV unless they acquire negative buoyancy by encapsulating heavy solutes, such as ribose, and descend from the water surface. We have also shown that some primordial water constituents, for example, ferric salts, can provide UV attenuation strong enough to protect liposomes at depths of several millimeters [27,28]. The process forms resilient, UV-protected, autocatalytic membranes; ensures liposomal survival, fusion, fission, and component mutation; and provides liposomes’ adaptation and persistence. The hypothesis comprises the necessary prerequisites for Darwinian evolution: mutable adaptive traits, heredity, and selective forces. A pictorial representation of the hypothesis is depicted in Figure 1.

## 3. A Model of the Origin of Life’s Homochirality

It is thought that on early Earth, pathways may have already been present to transform simple lipid (fatty acid) membranes into phospholipid membranes [29,30,31]. There is multiple evidence that lipid bilayers, including glycerophospholipid membranes, are chiral and enantioselectively permeable, even though the exact nature that remains elusive [1,32]. The most realistic explanation is that homochiral packing is energetically and structurally favored [33]. It was also experimentally demonstrated that homochiral membranes can originate from heterochiral lipid mixtures, due to cooperative packing and curvature, when one chirality can override the other [34]. There is also experimental evidence suggesting that phospholipid membranes preferentially favor ribose compared to other pentose compounds [35].

One of the sources of life’s homochirality, discussed in the literature, is the diastereoselective permeability of phospholipid membranes [32] and vesicles [35,36]. A significant aspect of that, demonstrated in Goode et al. [36], is that the membranes’ enantioselective permeability significantly favors d-ribose over l-ribose. Importantly, that preference does not depend on whether the membrane is d-Archaeal or l-Hybrid.

Note that the selective permeability for d-ribose has been studied only for enantiomerically pure membranes. Although prebiotic condensations of glycerol, phosphate, and fatty acids can produce phospholipid esters with a racemic backbone, which might affect the enantioselectivity of membrane permeability [37,38], no direct experimental evidence for this is currently known. In contrast, many experimental studies using vesicles as protocell models have solely employed commercial enantiopure phospholipids [37]. This choice may be due to the fact that all biological lipid bilayers, including glycerophospholipid membranes, are homochiral [1,2]. Future experiments, as suggested in Section 6, could address this research gap.

In addition to enantioselective permeability, the mechanism we propose is the phosphorylation of d-ribose into d-ribose-5-phosphate, and trapping and accumulation of the d-ribose-5-phosphate, converted within phospholipid membranes. We suggest that a phospholipid membrane can donate a phosphate group, ${\mathrm{PO}}_{4}^{2-}$, to a d-ribose passing through the membrane via a transphosphorylation reaction. A detailed illustration of a similar process can be found in [39]. Although there is no direct evidence for the proposed transfer of phosphates from phospholipids to ribose, our assumption relies on other instances of non-enzymatic phosphorylation, for example, phosphorylation of pyruvate [40]. This is justified because both molecules are polar and water-soluble, possess carbonyl C═O groups, and feature hydroxyl-related chemistry.

A phosphorylated d-ribose, after attaching a negatively charged phosphate group, cannot cross the hydrophobic lipid barrier and becomes trapped within the vesicle [41]. The proposed mechanism thereby breaks the racemic equilibrium and enables the next evolutionary step: converting trapped d-ribose-5-phosphate into complex functional molecules, such as ribozymes, RNA, and ultimately DNA.

In most circumstances, the transphosphorylation reaction is slow because it requires breaking strong P-O bonds in the phospholipid. However, in the presence of mineral catalysts, such as ${\mathrm{Fe}}^{3+}$, the phosphorylation rate can be significantly increased even in an aqueous environment [39,42,43]. The iron ${\mathrm{Fe}}^{3+}$ ions are components of ferric chloride ${\mathrm{FeCl}}_{3}$ formed via iron oxidation by chlorine. Ferric chloride and other iron-containing species were likely present in the prebiotic, early Earth environment, particularly through interactions among iron-rich minerals, volcanic activity, and volcanic outgassing of hydrochloric acid. Volcanic ash and meteoritic material brought significant amounts of metals, including iron, to the surface. Acidic reactions involving chlorides, common in volcanic environments, can form iron chlorides. The presence of ${\mathrm{Fe}}^{3+}$ was confirmed by analysis of sedimentary archives from the Archean ocean [44]. Additionally, ${\mathrm{Fe}}^{3+}$ could be produced by surface-mediated oxidation of ${\mathrm{Fe}}^{2+}$ adsorbed onto mineral surfaces (clays, oxides) [45]. Other minerals, like borate, also accelerate the phosphorylation rate [46].

**Figure 2:**
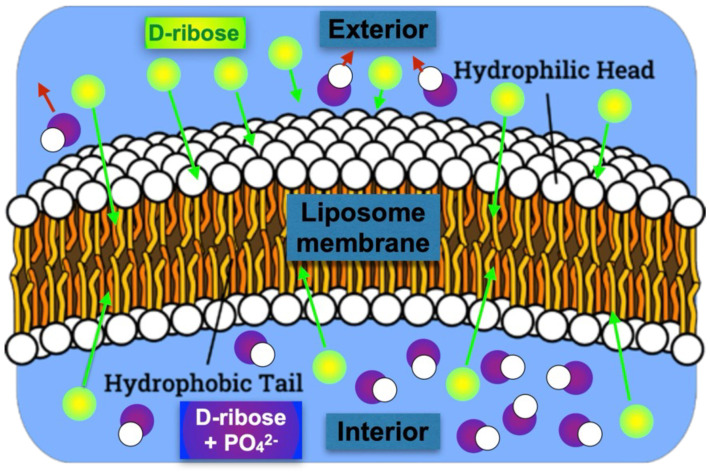
A simplified illustration of the model. It depicts a cross-section of a spherical phospholipid vesicle, highlighting its membrane with hydrophilic heads and hydrophobic tails. Racemic d- and l-ribose (green) can both penetrate the membrane and capture the PO groups, but d-ribose undergoes this process more quickly and is preferentially trapped (purple) inside the vesicle.

All of these accumulate d-ribose derivatives within the liposome, thereby increasing their concentration. We hypothesize that, due to enantioselective permeability and subsequent phosphorylation, the buildup of d-ribose occurs more rapidly than that of l-ribose. A simplified artistic illustration of the model is depicted in Figure 2.

The proposed concept—of (a) self-assembly of phospholipid liposomes in Archean waters containing a mixture of d- and l-ribose, (b) liposome submergence that provides protection against solar UV and at the same time is greatly enhanced by the presence of ${\mathrm{Fe}}^{3+}$ in Fe-rich Archean waters, and (c) the selective accumulation of phosphorylated d-ribose, also boosted by the presence of ferric salts—collectively creates a comprehensive and self-contained framework describing the origin of modern life’s chirality.

## 4. Simple Process Model

The process outlined in the previous section can be modeled by simple balance equations. Consider the following assumptions:

1. A spherical liposome containing a racemic mixture of d- and l-ribose at equal concentrations is immersed in a large prebiotic water pool containing the same racemic mixture at the same concentrations.
2. Ribose attaches a negatively charged phosphate group while passing through the membrane (phosphorylation) and becomes trapped inside the membrane by electrostatic forces.

In a simple case of symmetric in-out membrane permeability *P*, the total ribose flux per unit area through the membrane is

(1) $$I=\left(n_{out}-n_{in}\right)P,$$

where $n_{out}$ and $n_{in}$ are the ribose densities outside and inside the membrane, respectively. Clearly, in the absence of particles’ source or sink, the equilibrium condition I=0 requires equal densities $n_{in}=n_{out}$ on both sides, regardless of permeability. Therefore, if the initial ribose outside is racemic, it will remain racemic inside the membrane as well.

Now, include the process of capturing the phosphate group in the membrane. Assume, for simplicity, that the process of membrane penetration and the phosphorylation reaction can be expressed in terms of a reaction rate $K_{p}$. We present the rate $K_{p}$ as an effective pseudo-first-order rate constant, corresponding to the fraction *X* of a reagent converted over a time interval *T*,

(2) $$K_{p}=-ln\left(1-X\right)/T.$$

To estimate $K_{p}$, we use data published in [39], in which the effect of ${\mathrm{Fe}}^{3+}$ on ATP synthesis via phosphorylation of ATD was studied. Although not specific to d-ribose, it still provides some insight into the synergistic action of membrane penetration and PO group capture. In the study, 15% of ADT was converted to APT in approximately four hours, which, according to Equation (2), yields $K_{p}=1\times 10^{-5}s^{-1}$. This value should be interpreted as an order-of-magnitude estimate consistent with experimentally reported reaction times, rather than as a precise or universal kinetic parameter.

The probability Π of the reaction is

(3) $$\Pi =1-\exp\left(-K_{p}\delta /P\right),$$

where δ is the membrane thickness. For a small δ, such that $K_{p}\delta /P\ll 1$, the fraction of the flux that undergoes phosphorylation is $\Pi =K_{p}\delta /P$. To check this approximation, substitute $K_{p}=1\times 10^{-5}s^{-1}$, δ=5nm, and $P=1\times 10^{-7}\mathrm{cm}s^{-1}$ [35], which would yield $K_{p}\delta /P=5\times 10^{-5}<<1$.

The behavior of the non-phosphorylated density $n_{np}$ within the membrane can be described by

(4) $$V\frac{dn_{np}}{dt}=An_{out}P\left(1-\frac{K_{p}\delta }{P}\right)-An_{np}P,$$

where $V=4/3\pi R^{3}$ is the vesicle volume, $A=4\pi R^{2}$ is its surface area, and *R* is its radius. The first term on the RHS of Equation (4) represents the inward flux adjusted by the phosphorylation reaction. The second term is the outward flux. Clearly, under the assumption of $K_{p}\delta /P\;<<1$, the equilibrium non-phosphorylated inside density is very close to that outside $n_{np}\approx n_{out}$.

Assume further a perfect trapping of the phosphorylated d-ribose. Later, we expand on this assumption. The phosphorylated part of the inward flux determines the behavior of the trapped phosphorylated density within the membrane,

(5) $$V\frac{dn_{p}}{dt}=An_{out}K_{p}\delta .$$

Note here that not all the inward flux reaches the membrane’s interior. Some of it, after phosphorylation near the outer boundary, would be repelled outward by the electrostatic forces. However, by symmetry, this loss would be exactly balanced by phosphorylated outward flux converted near the inner boundary and repelled inward.

Considering all the points mentioned, the solution for the ribose density inside the membrane $n_{in}$ is the sum of the non-phosphorilated and phosphoralated densities $n_{in}=n_{np}+n_{p}$:

(6) $$n_{in}\left(t\right)=n_{out}\left(1+t/\tau_{d}\right),$$

where the characteristic “doubling” time τ is

(7) $$\tau_{d}=\frac{V}{AK_{p}\delta }=\frac{1}{3K_{p}}\frac{R}{\delta }.$$

As a numerical estimate, assume a typical membrane geometry of R=50nm, δ=5nm, and the volumetric reaction rate $K_{p}=1\times 10^{-5}s^{-1}$ resulting in a characteristic time $\tau_{d}=92h$. Therefore, after just under four days, the concentration of d-ribose inside the membrane will double.

To generalize Equation (5), include a loss term for the phosphorylated d-ribose and modify the equation accordingly:

(8) $$\frac{dn_{p}}{dt}=\frac{n_{out}}{\tau_{d}}-\frac{n_{p}}{\tau_{conf}},$$

where $\tau_{d}$ is defined by Equation (7) and $\tau_{conf}$ is a confinement time of a generic loss mechanism, combining all possible losses, for example, the membrane leakage, the ribose disassembly, etc. If each of these losses can be characterized by its own confinement times $\tau_{conf1},\tau_{conf2}\ldots$, then the overall confinement is determined by $1/\tau_{conf}=1/\tau_{conf1}+1/\tau_{conf2}\ldots \;$.

Note that the equilibrium phosphorylated d-ribose density is $n_{p}=n_{out}\tau_{conf}/\tau_{d}$, so the higher the ratio $\tau_{conf}/\tau_{d}$, the higher the final d-ribose density. Taking Equation (8) into account, a time-dependent solution for the d-ribose density inside the membrane is:

(9) $$\frac{n_{in}}{n_{out}}=\frac{\tau_{conf}}{\tau_{d}}-\left(\frac{\tau_{conf}}{\tau_{d}}-1\right)e^{-\frac{t}{\tau_{conf}}}$$

**Figure 3:**
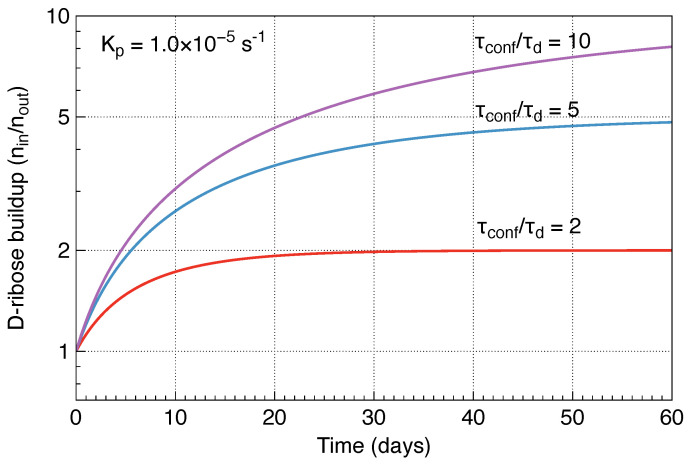
A time evolution of the D−ribose buildup factor $n_{in}/n_{out}$ plotted for a phosphorylation rate $K_{p}$ of $1\times 10^{-5}s^{-1}$ and different ratios $\tau_{conf}/\tau_{d}$.

Figure 3 illustrates the time evolution of the d-ribose buildup factor $n_{in}/n_{out}$. Each curve asymptotically approaches its equilibrium value $\tau_{conf}/\tau_{d}$, with the growth rate affected by the phosphorylation rate $K_{p}$.

## 5. Model Discussion

The above model clearly does not cover all potential scenarios or include every relevant physical and chemical detail. Instead, we intentionally simplified the problem to emphasize the key aspects, aiming to showcase a robust, promising, and self-contained scenario while providing an approximate estimate and bounds on the effect.

One of the peculiar consequences of our assumptions is the absence of the explicit presence of the membrane permeability in the growing rate of the phosphorylated component—Equation (7). The reason for that is the use of a single reaction rate parameter $K_{p}$ that only implicitly contains the dependence on the membrane permeability. A more accurate model that incorporates the surface localization of phosphorylation would undoubtedly restore the explicit permeability dependence and would only amplify the buildup of d–ribose within the membrane.

Another essential factor could be a process that would disrupt the linear buildup of d-ribose as described in Equation (6), such as imperfect trapping or other mechanisms. We estimated this effect by introducing a generalized loss mechanism and demonstrated that the buildup factor is proportional to the ratio of the mechanism’s confinement time to the phosphorylation time. The latter also determines the growth rate of d-ribose buildup.

We did not explicitly include l-ribose dynamics, as it would merely mimic those of d-ribose but with different governing parameter values, such as permeability and phosphorylation rate. As mentioned earlier, some studies have demonstrated that the d-ribose enantiomer generally has a higher membrane permeability. Whether the d-ribose has a higher phosphorylation rate than l-ribose and whether and to what extent this is relevant to our prebiotic scenario, remains unknown and, hopefully, could be assessed through experimentation. Again, note that even a modest imbalance (2-5x) in the initial concentrations of the two enantiomeric forms can be amplified over time and would be sufficient to drive enrichment.

## 6. Suggested Experimental Tests

Our hypothesis outlined above can be tested in the following experiments:

1. Liposomes with different membrane compositions are loaded with a racemic D/L ribose mixture. The key idea is that different membrane compositions will provide diverse tools for enantiomeric ribose separation [47,48].
2. Liposomes are suspended in a racemic ribose mixture.
3. Add varying concentrations of ${\mathrm{FeCl}}_{3}$.
4. The membrane composition that encourages d-ribose-5-phosphate accumulation in liposomes, leading to D’s predominance over L, would be an ideal candidate for further study into abiogenesis and the origins of life chirality.
5. Repeat the experiments above, this time with membranes made of racemic phospholipids.

## 7. Conclusions

We suggest a mechanism linking the emergence of life’s homochirality to the specific ways in which an initially racemic mixture of d- and l-ribose interacts with phospholipid membranes in bilayer vesicles. d-ribose, passing through the membrane, can bind to the membrane’s phosphate group and become trapped inside. The presence of ${\mathrm{Fe}}^{3+}$, a metal ion catalytic agent, could significantly increase the phosphorylation rate. Our model showed that, in just a few days, the concentration of d-ribose inside the membrane vesicle would double. We discuss key factors, including d-ribose phosphorylation rate and d-ribose loss. We acknowledge that some supporting experiments have been conducted in studies such as mineral-assisted phosphorylation or the permeability of plants’ membranes, rather than in the prebiotic environment we are describing. We hope the proposed experimental tests will shed further light on these critical issues.

We hypothesize that the enantioselective character of membrane permeability and possibly of the ribose phosphorylation results in the selective accumulation of phosphorylated d-ribose, leading to the formation of d-deoxyribose-5-phosphate. Both d-ribose and d-deoxyribose are crucial for establishing the chirality of d-RNA and d-DNA. In turn, the homochirality of d-deoxyribose and d-ribose nucleic acids determines the l-isoform of amino acids and proteins [2,10,49,50,51,52].

In summary, the proposed hypothesis organically integrates concepts proposed earlier: (a) the self-assembly of phospholipid liposomes in Archaean waters containing a mixture of d- and l-ribose, (b) liposome submergence that provides protection against solar UV, and (c) selective accumulation of phosphorylated d-ribose. The presence of ${\mathrm{Fe}}^{3+}$ boosts both UV protection and selective accumulation of phosphorylated d-ribose. Collectively, these elements form a comprehensive, self-contained framework that explains the origin of the chirality of modern life. 

## Data Availability

The original contributions presented in this study are included in the article. Further inquiries can be directed to the corresponding author.
